# Analysis of ignition and flame geometric characteristics of lubricating oil leaking from automotive engine onto hot surfaces

**DOI:** 10.1371/journal.pone.0319934

**Published:** 2025-03-21

**Authors:** Lei Bai, Changchun Liu, Liubing Wang

**Affiliations:** 1 College of Safety Science and Engineering, Xi’an University of Science and Technology, Xi’an, China; 2 Xi’an Key Laboratory of Urban Public Safety and Fire Rescue, Xi’an, China; GH Raisoni College of Engineering and Management Pune, INDIA

## Abstract

The ignition and combustion process of lubricating oil leaking from an automotive engine onto a hot surface is a major cause of vehicle fires, and the geometric characteristics of the flame directly affect the spread and severity of the fire. Therefore, studying the ignition characteristics of lubricating oil on hot surfaces and quantifying flame behavior is of great significance for vehicle fire safety protection. This study utilizes a self-developed automotive hot surface ignition oil simulation platform, employing the SOBEL threshold segmentation algorithm combined with box-counting fractal dimension theory. It investigates the factors affecting the ignition delay time of automotive engine lubricating oil, the ignition risk and probability on engine hot surfaces, and analyzes the temporal evolution characteristics of the flame fractal dimension of engine lubricating oil. This research provides theoretical support for vehicle fire risk assessment and prevention. The main findings of this study are as follows: (1) As the temperature of the hot surface increases, the ignition delay time generally shows a decreasing trend, with 450°C being a critical turning point; (2) There is an overlap between ignition and non-ignition cases within a specific range, forming a possible ignition zone, and the *R*² values of the fitting equations for the upper and lower boundaries are both above 95%, indicating a good fit. (3) The fractal dimension can effectively quantify the geometric complexity of the flame’s outer contour, thereby characterizing the stability of the flame’s combustion. The evolution of the fractal dimension of the lubricating oil droplet flame shows a trend of first increasing and then slowly decreasing. The interval from 0 to 1 second is the stable combustion phase, from 2 to 3 seconds is the unstable combustion phase, and from 3 to 5 seconds is the secondary stable combustion phase. During this period, the fractal dimension gradually decreases from the peak to around 1, and the flame’s outer contour transforms from complex to simple. (4) The volume of the droplet (*V*) affects both the peak value of the fractal dimension (*D*_*max*_) of the flame and the time at which it occurs (*t*_*max*_). The larger the volume, the earlier *D*_*max*_ occurs. For a 0.1 ml droplet, *D*_*max*_ occurs earliest (*t*_*max*_ = 1.98 s), while for a 0.5 ml droplet, *D*_*max*_ appears the latest (*t*_*max*_ = 3.22 s). There is a significant correlation between *t*_*max*_ and droplet volume *V* (*R* = 0.995, *P* = 0.001). The spray hole size has a greater impact on *D*_*max*_ compared to *t*_*max*_. With spray hole diameters ranging from 0.4 mm to 0.7 mm, the fractal dimensions of all droplet flames appear at around 2.6 seconds, but the values of *D*_*max*_ vary significantly. As the spray hole diameter (*S*) decreases, *D*_*max*_ approaches 2. When the spray hole diameter is 0.4 mm, *D*_*max*_ is the highest, reaching 1.605, indicating the most drastic change in the geometric complexity of the flame’s outer contour and the least stable combustion process overall.

## Introduction

Leakage of lubricating oil from automotive engines is a potential hazard [1,2]. When lubricating oil leaks from the oil pan gasket or when deformation or rupture of the oil pipeline or container leads to droplet leakage that falls onto the exhaust pipe, it can easily cause fires or even explosions [3,4]. The ignition of droplets on hot surfaces is not only closely related to the surface temperature but also influenced by factors such as droplet volume and size. The ignition process of lubricating oil droplets has high uncertainty [5,6], and scientific research is urgently needed to clarify its influencing factors and risks. In addition, the geometric characteristics of the flame during combustion play a significant role in the spread and stability of combustion [7,8], while the variation in the fractal dimension of the flame can reveal the complexity and dynamic evolution of the flame interface [9,10]. This paper focuses on the ignition behavior and flame geometric characteristics of leaked fuel from automotive engines on hot surfaces, aiming to provide theoretical support and practical guidance for fire prevention and control of fuel leaks in engines.

The process and mechanism of liquid fuel ignition on hot surfaces are complex, involving droplet boiling, diffusion, evaporation, and other processes. Previous researchers have conducted extensive studies and analyses on the ignition mechanism of hot surfaces. Volkov et al. [11] studied the main factors and mechanisms affecting heat and mass transfer on hot plates. Through the developed mathematical model of heat and mass transfer, they established a combustion mode of air-vapor mixtures of liquid fuels ignited by localized heating. Stouffer et al. [12] investigated the effect of fuel and air temperature on fuel ignition performance, showing that ignition performance for most fuels is closely related to viscosity and distillation properties. Mevel et al. [13] found that the chemical properties of the mixture, surface properties (e.g., geometry and material), and heating rate determine the minimum temperature required for thermal surface ignition of reactive gases, as well as the thermal non-uniformity of the hot surface. Boeck et al. [14] used experiments combined with numerical simulations to study the hot surface ignition dynamics of premixed hydrogen and air. Their numerical results indicated multiple combustion transients within the thermal boundary layer following the initial ignition event, with ongoing chemical reactions in the thermal plume above the hot surface during the later stages of combustion. Zhang S et al. [15] analyzed the thin-film evaporation of spherical droplets on hot surfaces, refining Stimson’s classical flow field solution. Bennett et al. [16] analyzed three distinct boiling modes (i.e., nucleate, transition, and film boiling) of droplets on hot surfaces. Gulder et al. [17,1] proposed an analytical model for the evaporation and ignition of spherical fuel droplets on hot surfaces.

In recent years, the study of hot surface ignition characteristics has gradually become a key focus in the field of vehicle fire safety. Colwell et al. [18] showed that hot surface ignition is inherently a probabilistic event, which cannot be defined by a single ignition temperature. Their study clarified the relationship between surface temperature and ignition probability. Although it provided a comprehensive review of hot surface ignition testing, it is difficult to extrapolate the results to the fundamental physical and chemical processes leading to hot surface ignition. Kuchta et al. [19] investigated the relationship between the hot surface ignition temperature of automotive fuels, such as engine lubricating oil, and the hot surface area, but their experiment did not consider the effect of surface geometry or orientation. Davis et al. [20] analyzed the impact of fuel chemical properties on the hot surface ignition temperature, finding that the temperature at which the ignition probability is 50% is closely related to the hot surface ignition temperature. In 2010, Davis et al. [21] used the same experimental setup to study the ignition phenomena of flammable liquids like leaded gasoline, nitromethane, and methanol on 304 stainless steel hot surfaces, discovering that the hot surface ignition temperature is influenced by the structure of the fuel. They found that the ignition temperatures of gasoline and methanol were consistent with previous studies, being hundreds of degrees Celsius higher than the autoignition temperature of the liquids. Goyal et al. [22] measured the hot surface ignition temperature of n-heptane, turbine engine fuels (Jet-A, JP-8, JP-5), piston engine fuels, and low-lead aviation gasoline. Their research showed that hot surface ignition temperatures are always higher than autoignition temperatures, and hot surface ignition at high temperatures is a probabilistic event. Lapointe et al. [23] studied the influence of gasoline octane number on hot surface ignition characteristics under different climate conditions. Byers K et al. [24] examined the risk of igniting leaked gasoline on hot surfaces in engine compartments and exhaust systems. Mitu et al. [25] proposed an empirical model to estimate the minimum hot surface ignition temperature for aviation kerosene, but did not consider the impact of radiative heat transfer. Other researchers have used micro-syringes, combined with thermocouples and high-speed cameras, to analyze the entire process from droplet falling to evaporation and ignition of ethanol [26–28].

In the aforementioned studies on ignition characteristics, the minimum hot surface ignition temperatures of flammable liquids were consistently higher than their respective autoignition temperatures. The majority of research has focused on gasoline, diesel, and pure fuel substances, while studies on automotive non-fuel liquids like engine lubricating oil are relatively scarce. Due to its high viscosity, leaked engine lubricating oil droplets tend to adhere to hot exhaust pipes [29], making them as hazardous as gasoline leaks. However, there is little research on the ignition characteristics of lubricating oil droplets of different sizes. Additionally, studies on the geometric complexity of flames during oil droplet combustion are limited. The geometric complexity of a flame refers to the complex spatial structures that appear during combustion, including irregular shapes, fractal structures, wrinkles, and deformations caused by turbulence, all of which reflect the interactions between chemical reactions and fluid dynamics during the combustion of oil droplets [30,31]. In hot surface ignition of oil droplets, analyzing flame geometric complexity is crucial for a deeper understanding of the combustion mechanism, including heat release, mixing, and mass transfer processes, and how these factors influence combustion efficiency and stability [32,33].For vehicle fire prevention and control, analyzing flame geometric complexity can help predict flame propagation behavior, provide scientific support for designing efficient fire suppression systems, selecting appropriate fire extinguishing agents, and establishing safety protocols, thereby enhancing the effectiveness of fire prevention and control. Although some researchers have discussed flame height and width for droplets of different sizes [34,33] and compared the flame area under different chemical compositions [35,36], these parameters can only reflect the overall shape of the flame and cannot quantify the boundary complexity and irregularity of the flame. This neglects local dynamic characteristics, has limited applicability to highly irregular or asymmetric flames, and fails to fully reveal the geometric characteristics of complex combustion processes. Up to now, there has been little research on the temporal evolution characteristics of the fractal dimension of flames from flammable droplets.

Based on the above research background, this study uses fully synthetic engine lubricating oil as the base material, with a self-developed automotive hot surface ignition simulation platform as the experimental setup, combined with high-speed imaging techniques. Initially, three key factors were studied: hot surface temperature (*T*), droplet volume (*V*), and spray hole diameter (*S*), and their effects on ignition delay time and ignition probability were analyzed. The distribution characteristics of three ignition zones—safe zone, possible ignition zone, and inevitable ignition zone—were determined. Subsequently, the SOBEL threshold segmentation algorithm and image binarization were used to extract the flame’s outer contour at different time points. The box-counting-based fractal dimension algorithm was applied to study the evolution of flame geometric complexity. The study ultimately obtained the evolution pattern of the fractal dimension (*D*) of droplet flames with varying droplet volumes (0.1–0.5 ml) and spray hole diameters (0.4–0.7 mm). This research is of great significance for fire studies involving liquid fuel combustion, such as vehicle fires, as it provides a scientific basis for predicting the geometric characteristics of flame propagation during combustion and helps assess fire risk more accurately.

## Theory & methods

During combustion, flames typically exhibit highly complex and dynamically changing shapes, with their geometric characteristics being the result of the combined effects of turbulence, chemical reactions, heat transfer, and mass transfer [37]. Due to the nonlinear and multi-scale nature of these influencing factors, the flame boundary often displays irregular geometric patterns that are difficult to accurately characterize using traditional geometric description methods [38]. This irregularity raises the requirements for studying flame propagation characteristics and combustion stability. Fractal dimension can quantify the complexity and detail of irregular geometric shapes, and compared to traditional geometric description methods, it more accurately reflects the geometric characteristics of flame boundaries at different scales [39]. Numerous studies have shown that there is a close relationship between the fractal dimension of a flame and its combustion stability. A higher fractal dimension usually indicates greater complexity at the flame boundary, reflecting instability in the combustion process [40,41]. Therefore, calculating the fractal dimension of a flame can provide important insights for evaluating flame stability and formulating control strategies.

Among the methods for calculating fractal dimension, the box-counting method is the most widely used due to its clear concept, simple calculation process, and suitability for various irregular shapes [42]. The core principle is to embed the object of study into a grid and count the minimum number of grids required to cover the object at different scales, thereby establishing a logarithmic relationship between grid size and the number of grids covering the object. This relationship is used to calculate the fractal dimension. The box-counting method lays a mathematical foundation for analyzing the correlation between flame morphology and combustion performance.

In this study, the colored flame images consist of three channels (RGB), each containing different pixel values. In certain areas, the red channel may exhibit significant gradient changes, while the green or blue channels may show less variation, making it challenging for the computer to accurately identify the actual flame boundary and thereby affecting the precise calculation of the flame’s fractal dimension. Therefore, binarized flame images were used to calculate the fractal dimension.

First, the SOBEL threshold segmentation algorithm is used to extract the flame edge contour, employing two 3 × 3 convolution kernels to calculate the gradients in the horizontal (*x*-direction) and vertical (*y*-direction) directions, respectively:

Horizontal Gradient Detection *G*_*x*_:


$$K_{x}=\left[\begin{array}{ccc} -1 & 0 & 1\\ -2 & 0 & 2\\ -1 & 0 & 1 \end{array}\right]$$ (1)

Convolve *K*_*x*_ and *K*_*y*_ with the input image separately to obtain the gradient images in the *x* and *y* directions. For each pixel position (*i*, *j*), cover that pixel and its surrounding 3 × 3 region with the convolution kernel, and calculate the weighted sum:


G(i,j)x=∑u=−11∑v=−11Kx(u+1,v+1)·I(i+u,j+v) (2)

Where *I* (*i*, *j*) represent the grayscale value of the original image.

Based on the horizontal and vertical gradients, calculate the gradient magnitude of each pixel. The gradient magnitude reflects the intensity of the grayscale change, with larger values indicating more pronounced edges:


$$G(i,j)=\sqrt{G_{\left(x\right)}{\left(i,j\right)}^{2}+G_{\left(y\right)}{\left(i,j\right)}^{2}}$$ (3)

Then, by setting a threshold, weak edges are filtered out, leaving only strong edges.

**Fig 1:**
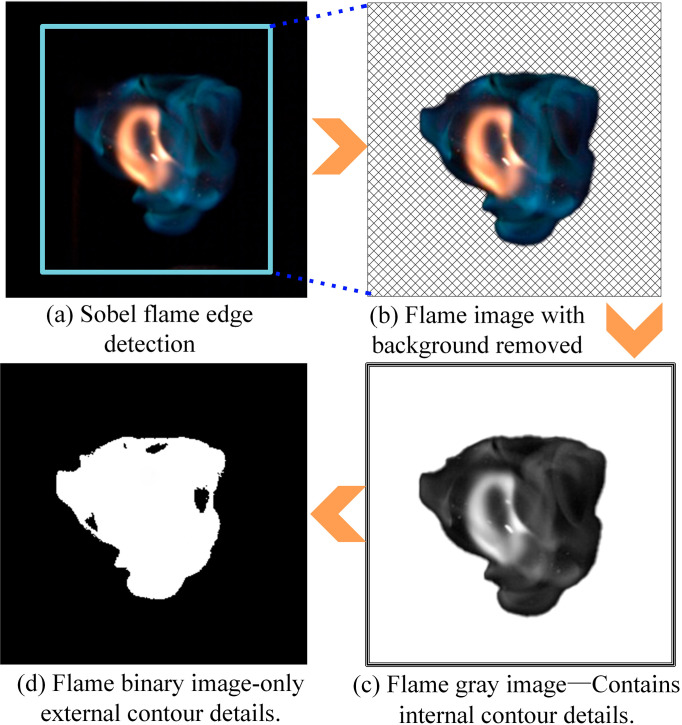
Flame binarization image processing flow.

After converting the extracted flame contour into a grayscale image, the grayscale is adjusted to convert it into a binarized flame image by setting a black threshold. The specific process is shown in Fig 1.

**Fig 2:**
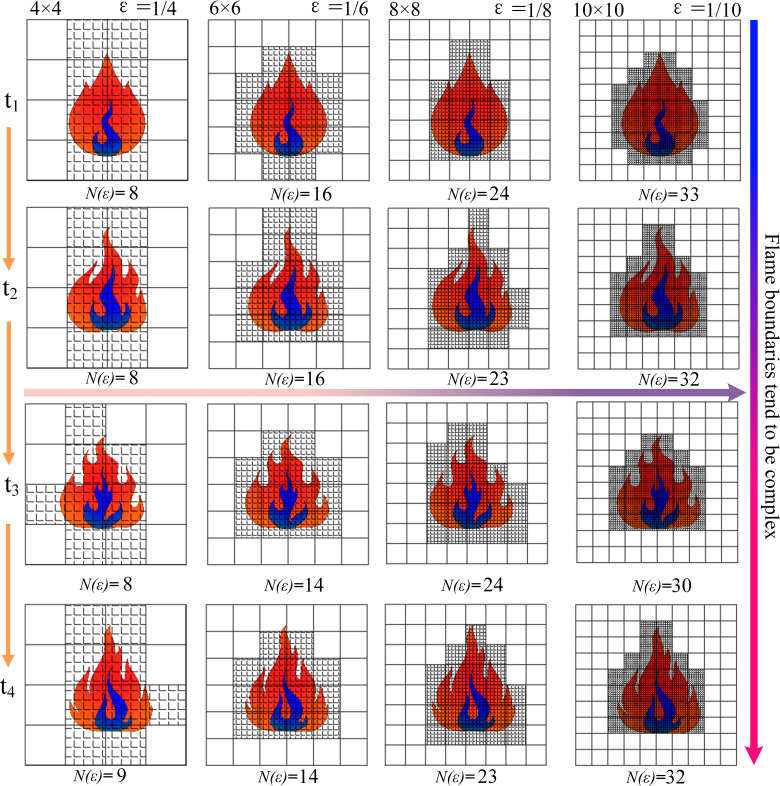
Fractal dimension of flame based on box counting method.


After obtaining the binarized image of the flame contour, the box-counting grid is constructed. A set of square grids is overlaid on the flame contour image. Initially, the grid scale (side length) is large, and then the scale is gradually reduced. As shown in Fig 2, the grid becomes smaller and more numerous from left to right. For each specific scale *r* of the grid, count the minimum number of grids *N*(*r*) required to cover the flame contour (if a grid contains any part of the flame contour, it is considered occupied). As the grid scale decreases, more smaller grids are needed to cover the flame contour, as shown in Fig 2. The fractal dimension is then calculated according to Eq. 6:

$$D=\underset{\varepsilon \to 0}{\lim}\frac{\log N(\varepsilon )}{\log(\frac{1}{\varepsilon })}$$ (4)

Where *N*(*ε*) is the minimum number of boxes needed to cover the flame contour, and *ε* is the side length of the box (grid scale). *D* represents the fractal dimension, which ranges between 1 and 2. A value closer to 1 indicates that the flame boundary is closer to a smooth line, while a value closer to 2 indicates a more complex and irregular boundary (greater geometric complexity of the flame’s outer contour).

## Experiment

### Experimental materials

According to previous studies, automotive engine lubricating oil is relatively easy to ignite on a hot surface under stable evaporation conditions and sufficient oxygen availability. When a leak occurs and the oil comes into contact with a scorching exhaust pipe, it is highly likely to result in hot surface ignition, potentially causing vehicle fires. This risk should not be underestimated [43]. However, related studies remain relatively scarce up to now. Therefore, this study uses engine lubricating oil as the primary experimental material. The main components of lubricating oil are base oils and additives, where the base oil determines the primary performance of the lubricating oil, and additives are used to address its deficiencies. Considering factors such as low-temperature resistance, low volatility, and thermal stability, and to avoid experimental variability and errors caused by engine lifespan and operating temperature, the lubricating oil used in this study is new, unused, full synthetic oil, specifically 0W-40, which is widely used in the automotive manufacturing industry. The corresponding physical parameters are shown in Table 1.

**Table 1:** Physical parameters of engine lubricating oil.

Attribute	Result	Unit	Analytical method
**Kinematic viscosity (100°C)**	13.420	mm^2^/s	ASTM D445
**Kinematic viscosity (40°C)**	74.190	mm^2^/s	ASTM D445
**Viscosity index**	180	//	ASTM D2270
**Dynamic viscosity (-35°C)**	6125	mpa.s	ASTM D5293
**Density (15°C)**	839.3	kg/m3	ASTM D4052
**Flash point**	230	°C	ASTM D92
**Pour point**	-45	CEL	ASTM D97
**Calcium content**	0.147	%(m)	ASTM D6481
**Magnesium content**	0.110	%(m)	ASTM D6481
**Zinc content**	0.104	%(m)	ASTM D6481
**Phosphorus content**	0.089	%(m)	ASTM D6481
**Molybdenum content**	93	mg/kg	ASTM D5185

### Experimental instruments

**Fig 3:**
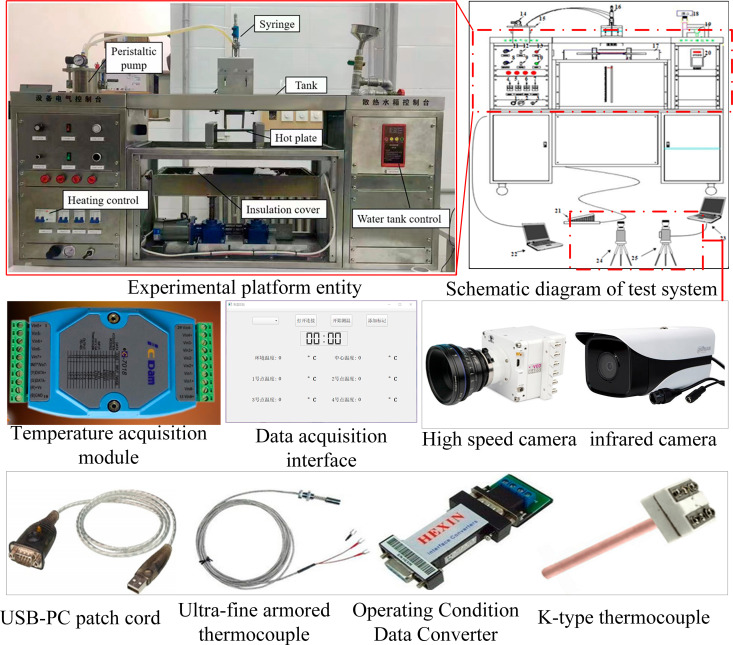
Experimental bench and corresponding facility.

The entire experiment in this study was conducted on a self-developed automotive hot surface ignition simulation platform. This platform consists of four main system modules: an image acquisition system, a hot surface heating system, an oil droplet simulation system, and a data acquisition system. The composition of the experimental system is shown in Fig 3. The image acquisition system comprises a Phantom® VEO640 high-speed camera and a computer for capturing video information of the combustion process. The high-speed camera features a 6000 Gpx/s global shutter and a sensor with 4 million effective pixels. It is equipped with a Canon 100 mm fixed-focus, image-stabilized lens. The video recording frame rate for this experiment was 600 fps, with an exposure time of 600 μs, and each image has a resolution of 1024 × 768 pixels, meeting the requirements for data analysis. The image measurement error is one pixel, corresponding to a size of 0.025 mm, and characteristic parameters of the combustion process were obtained through scale and pixel analysis. The infrared camera used is an FT1F thermal imaging camera with a sensor resolution of 4 million pixels, a maximum infrared aperture of F1.2, and a temperature measurement range of -20°C to 1500°C. The measurement accuracy is ± 5°C, and all parameters meet the requirements of this experiment. In the hot surface heating system, silicon molybdenum rods serve as the primary heat source, capable of reaching 1200°C under sealed conditions. The energy radiated by the silicon molybdenum rods can heat a 304 stainless steel plate (the most commonly used material for automotive exhaust pipes) to 670°C. The oil droplet simulation system consists of a pressure vessel, air pump, circuit board, custom solenoid valve, automatic dropper, foot switch, and pressure regulator, which can dispense oil droplets at a fixed location with a minimum droplet volume of 0.2 μl. The data acquisition system includes four TT-K-30-SLE K-type thermocouples for measuring the temperature of the hot plate surface, with a temperature range of 0–1150°C, a response time of 0.5 ms, a diameter of 1 mm, and an allowable deviation of less than 1.3°C. In addition, eight BNC-X-K type fast-response thermocouples are suspended 2 cm below the constant-temperature ceiling to measure the ambient temperature between the 304 stainless steel plate and the ceiling.

### Experimental plan and procedure

**Fig 4:**
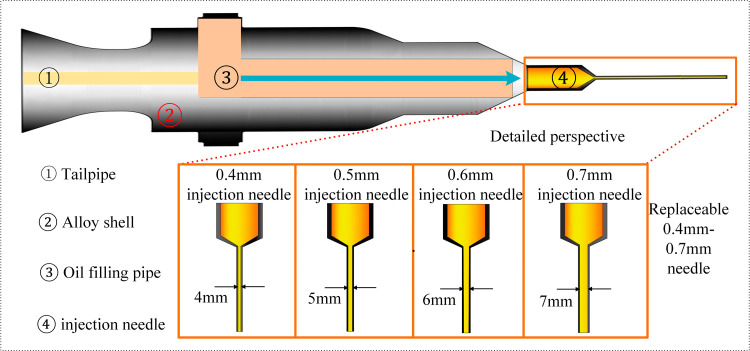
Schematic diagram of experimental injector size.

Based on previous studies, the exhaust pipe temperature of a vehicle ranges from 300–400°C under normal driving conditions and 400–500°C during high-speed driving. Additionally, 75°C is the typical temperature for lubricating oil under normal engine operating conditions, and at this temperature, the oil maintains good fluidity, effectively preventing blockage of the nozzle. Therefore, this experiment selected 300–500°C as the experimental temperature range, with repeated experiments conducted within this range to eliminate random errors [44,45]. The oil temperature was controlled at 75°C using a resistance wire. Most current hot surface ignition experiments for liquids involve single-drop experiments with fixed-volume liquid fuel (i.e., in a static state, a single droplet of liquid is dropped onto a horizontal hot plate to determine the ignition potential of various automotive and aviation liquids). However, studies on hot surface ignition experiments with different oil volumes are relatively rare [46]. Additionally, droplet size is an important parameter affecting the hot surface ignition delay time. Generally speaking, smaller droplets have a larger surface-area-to-volume ratio, which allows flammable droplets to absorb heat and evaporate more quickly, thereby shortening the time needed to reach a flammable vapor concentration and accelerating the ignition process. To better understand the ignition delay time on different hot surfaces, the needle of the fuel injection system in the experiment was replaced, as shown in Fig 4. Under the same pressure, the larger the needle’s inner diameter, the larger the size of the liquid droplets falling onto the hot surface. To prevent jet formation, needles with an inner diameter of less than 0.75 mm (0.4 mm, 0.5 mm, 0.6 mm, 0.7 mm) were used in the experiment to study the relationship between droplet size and hot surface ignition delay time [47,48]. In summary, this study conducted hot surface ignition experiments on lubricating oil within an experimental temperature range of 300–500°C, using a fixed spray hole diameter (0.4 mm) and fixed droplet volume (0.1 ml) to investigate the effects of droplet size and volume on ignition characteristics. The corresponding experimental conditions are shown in Tables 2 and 3.

**Table 2:** Experimental Conditions of Fixed Spray hole diameter.

experimental subject	Oil volume ml	Hot surface temperature °C	Spray hole diameter mm	ambient temperature °C	Number of experimental groups	Number of experiments
Engine lubricating oil	0.1	300-500 (5°C as a group)	0.4	18-24	40	400
	0.2		0.4	18-24	40	400
	0.3		0.4	18-24	40	400
	0.4		0.4	18-24	40	400
	0.5		0.4	18-24	40	400

**Table 3:** Experimental condition of fixed oil drop volume.

experimental subject	Oil volume ml	Hot surface temperature °C	Spray hole diameter mm	ambient temperature °C	Number of experimental groups	Number of experiments
Engine lubricating oil	0.1	300-500	0.4	18-24	40	400
	0.1		0.5	18-24	40	400
	0.1		0.6	18-24	40	400
	0.1		0.7	18-24	40	400

The specific steps of this experiment are as follows: First, connect the thermocouples and high-speed photography system to the remote computer, check the sealing of the oil pipe, and verify the coolant level in the water tank. Then, place the oil sample to be tested in the preheated pressure tank and clean the surface of the heated plate with a wire brush. Next, turn on the air pump, precisely adjust the oil drop volume to the present value using a pressure valve and micrometer, and mount the automatic dropper onto the support bracket (After the experiment, the droplet diameter was controlled by replacing the nozzle of the dropper (0.4, 0.5, 0.6, and 0.7 mm), as shown in Fig 4). Set the parameters and position of the high-speed camera, and record the location of the scale within the field of view. Subsequently, activate the heating platform and water tank heating device, raise the heating platform to make full contact with the heated plate. Monitor the temperature changes of the heated plate and surrounding area recorded by the thermocouples in real-time. When the temperature approaches the set value, trigger the high-speed camera to capture the target area, and record the laboratory temperature and humidity. Then, quickly lower the heating platform using the left-side electrical control panel, completely exposing the heated plate within the camera’s field of view. After turning off the heating platform, press the foot switch to activate the oil droplet and temperature acquisition system. This trigger action leaves a mark in the temperature data to facilitate reading of the instantaneous temperature. After the experiment ends, manually stop the video recording and temperature data collection. Turn on the fan to purge the experimental area of residual oil vapor, and use a wire brush of specific specifications to clean the surface of the heated plate. Finally, save the experimental video and temperature data, and check for any flame occurrence: if the oil droplet did not ignite, record the droplet volume and trigger temperature.

## Results

### Analysis of factors affecting ignition delay time of engine lubricating oil

**Fig 5:**
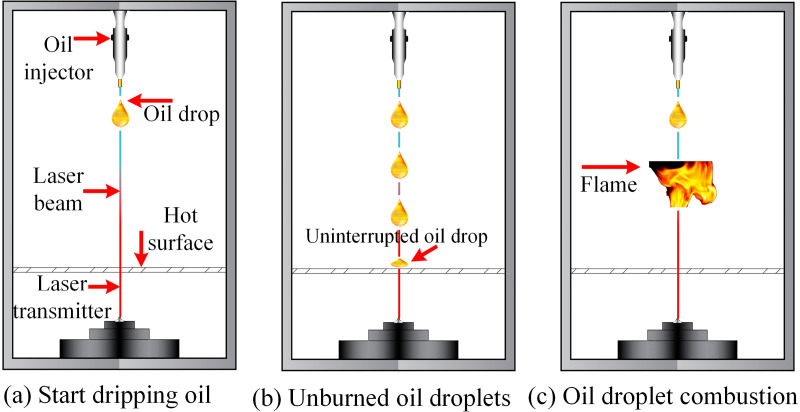
Ignition delay process shot by high-speed camera.

The ignition delay time reflects the time required for lubricating oil to accumulate and reach the ignition point on a heated surface. In this experiment, a high-speed camera was used to capture and store ignition events, with the experiment conducted under dark conditions (using a large aperture, high shutter speed, and low sensitivity mode to prevent loss of high-light details in the flame). A laser was projected directly below the oil spray hole. Once the experimental oil begins to spray, the laser shines on the oil droplet, forming a bright spot to determine the start of the oil dripping. The appearance of the bright spot marks the start of oil spraying in the experiment. The ignition delay time was determined by calculating the number of video frames from the moment the bright spot appeared until the flame was produced, as shown in Fig 5. The study ultimately obtained the evolution pattern of ignition delay time on hot surfaces for lubricating oil under different hot surface temperatures, droplet volumes, and spray hole diameters, as shown in Figs 8–10.

**Fig 6:**
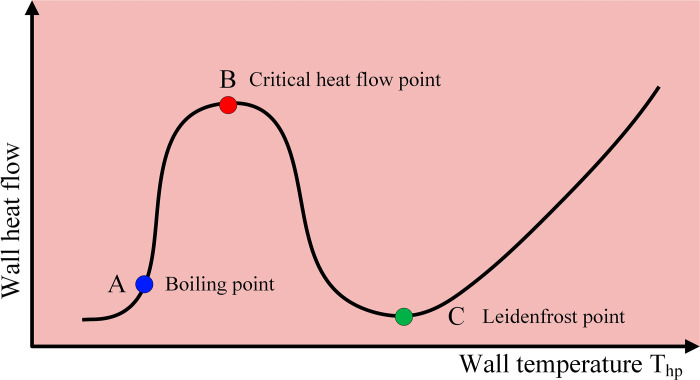
Variation of heat flow with wall temperature.

**Fig 7:**
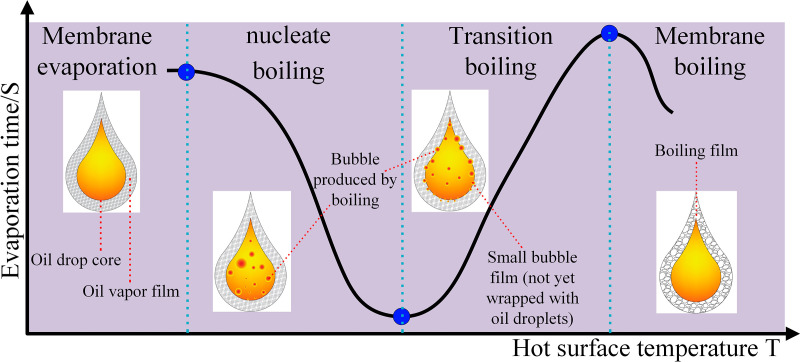
Droplet boiling modes at different temperatures.

### Influence of temperature on ignition delay time

The ignition delay time of engine lubricating oil is mainly influenced by the evaporation process [49], and ambient temperature and droplet temperature are the key factors affecting the evaporation process.

When the hot surface temperature changes, the heat transferred to the liquid also changes, which leads to different evaporation modes and evaporation rates for the fuel droplets falling onto the hot plate. The variation of the heat flux with hot surface temperature is shown in Fig 6, and the variation of the droplet and its boiling mode with hot surface temperature is shown in Fig 7.

When combustible liquid droplets fall onto a hot surface, four distinct boiling modes can be observed as the surface temperature increases: film evaporation, nucleate boiling, transition boiling, and film boiling [50]. When the hot surface temperature (THP) is below point A, and heating does not cause the droplets to boil immediately, a simple wetting mode occurs. In the range A < THP < B, during the film evaporation mode, the droplet maintains good contact with the hot surface, allowing high-speed heat transfer into the droplet. This results in vapor bubbles growing inside the droplet, leading to the rupture of the liquid surface and occasionally ejecting tiny droplets. As the temperature continues to rise, in the range B < THP < C, there is sufficient heat transfer between the droplet and the hot plate, enabling the droplet to lift off due to steam generation(During the contact between the high-temperature hot surface and the lubricating oil droplet, as the temperature continues to rise, particularly in the range of B < THP <  C, the heat transfer from the hot surface to the droplet gradually increases. The light components in the lubricating oil rapidly evaporate upon heating, and the liquid at the contact point between the droplet and the hot surface undergoes accelerated vaporization, forming a high-temperature steam film (known as the Leidenfrost effect). The formation of this steam film causes the steam to expand outward, generating a reactive force that gradually separates the droplet from the hot surface, leading to the observed “levitation” phenomenon of the droplet). This bouncing process is also accompanied by the ejection of small droplets, which is referred to as the transition boiling mode. At THP = C, the droplet reaches the Leidenfrost temperature. When the falling droplet approaches the substrate, a vapor cushion forms beneath it due to liquid evaporation. This vapor cushion acts as a lubricating layer and reduces energy dissipation during the spreading and recoiling of the droplet. When the wall temperature THP > C and the vapor layer is stable enough, the impacting droplet can rebound as a whole, which is referred to as the film boiling mode.

**Fig 8:**
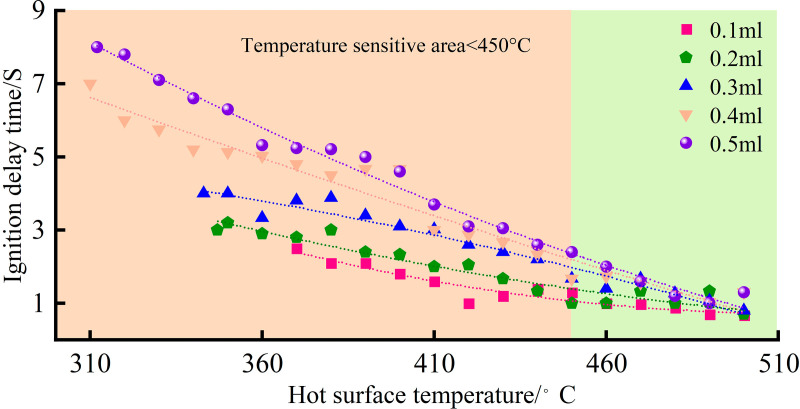
Relationship between hot surface temperature and ignition delay time.

Different boiling modes result in varying amounts of energy transferred from the hot surface to the droplet, affecting the evaporation rate of the droplet and the concentration of fuel vapor, thereby influencing the ignition delay time. Based on experimental data analysis, the relationship between the hot surface temperature and ignition delay time is obtained, as shown in Fig 8 (Table 4).

**Table 4:** Average ignition delay time in each temperature range.

Hot surface temperature range	300-350°C	360-400°C	400-450°C	400-500°C
Average ignition delay time/s(0.1-0.5ml)	5.71	3.73	2.15	1.19

As shown in Fig 8, the ignition delay time generally decreases with increasing temperature. When the hot surface temperature reaches 450°C, the rate of change in ignition delay time alters. Below 450°C, the temperature has a significant impact on ignition delay time, leading to a rapid decrease and notable differences in ignition delay times between different oil volumes. Within the temperature range of 450–500°C, the rate of decrease in ignition delay time gradually slows down. For an experimental oil volume of 0.1 ml, the ignition delay time ranges from 0.68 to 2.48 seconds within the ignition temperature range. For 0.2 ml of oil, the ignition delay time ranges from 0.71 to 3.18 seconds, with a faster decrease in ignition delay time compared to 0.1 ml as the temperature increases. At an oil volume of 0.3 ml, the ignition delay time decreases from 4 seconds to 0.8 seconds as the hot surface temperature rises. For oil volumes of 0.4 ml and 0.5 ml, the ignition delay time ranges from 1.03 to 7 seconds and 1.01 to 8.2 seconds, respectively. As the hot surface temperature increases, the difference in ignition delay time between these two volumes gradually diminishes. When the hot surface temperature exceeds 450°C, the ignition delay times become more similar, and the rate of decrease slows down.

### Influence of oil volume on ignition delay time

**Fig 9:**
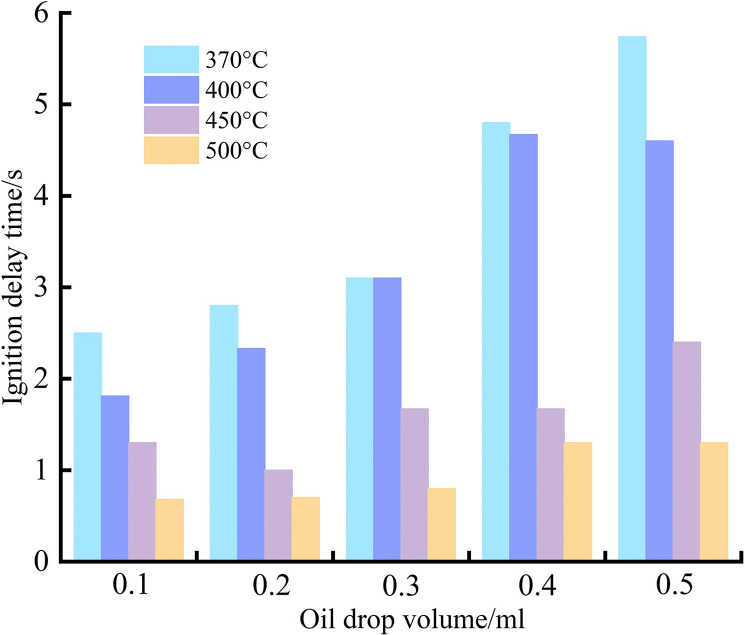
Relationship between fuel quantity and ignition delay time.

As shown in Fig 9, the experimental single-injection oil volume has a significant correlation with ignition delay time. At a hot surface temperature of 370°C, the relationship between oil volume and ignition delay time is positively correlated. At a hot surface temperature of 400°C, when the single-injection oil volume is 0.1–0.3 ml, the ignition delay time shows an increasing trend. For oil volumes of 0.4 ml and 0.5 ml, the ignition delay times are similar, ranging between 4–5 seconds. At hot surface temperatures of 450°C and 500°C, the ignition delay time shows only minor variations with changes in single-injection oil volume, fluctuating around 0.68–1.3 seconds.

The single-injection oil volume primarily affects ignition delay time by altering the concentration of the combustible mixture. From the perspective of boiling modes, before the hot surface temperature reaches 450°C, engine lubricating oil may exist in both film evaporation and nucleate boiling states. The smaller the oil volume, the shorter the time required for complete transition to the nucleate boiling state. This is because smaller oil volumes allow more energy to be transferred from the hot surface, accelerating the evaporation rate of the droplets. Consequently, more vapor molecules enter the air from the droplet surface, increasing the vapor concentration above the hot surface. Therefore, under the same temperature, an increase in oil volume results in a longer ignition delay time. Additionally, from an energy perspective, for the same temperature, smaller oil volumes result in more pronounced effects of absorbed energy on molecular motion within the droplet, leading to a higher evaporation rate. Thus, as oil volume increases, the ignition delay time tends to increase. When the temperature is in the range of 450–500°C, the influence of oil volume on ignition delay time diminishes. This is because, in this temperature range, the evaporation rate of droplets is generally high, and the time required for the vapor concentration to reach ignition conditions becomes similar, resulting in smaller differences in ignition delay time (Table 5).

**Table 5:** Global average ignition delay time of oil droplets with volume of 0.1-0.5ml.

Oil volume/ml	0.1	0.2	0.3	0.4	0.5
Average ignition delay time/s	1.57	1.70	2.16	3.11	3.51

### Effect of droplet size on ignition delay time

**Fig 10:**
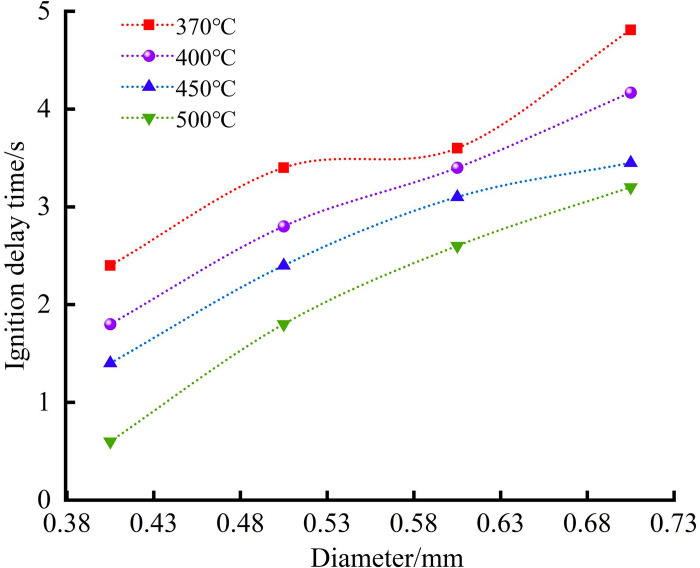
Relationship between ignition delay time and spray hole diameter.

Previous studies have shown that when the hot surface temperature *T*_*W*_ exceeds the Leidenfrost temperature, the droplet lifetime exhibits a negative correlation with *T*_*W*_. However, research by Xiong TY et al. [51] indicates that as *T*_*W*_ increases, the reduction rate of droplet lifetime slows down for smaller droplets and may even remain unchanged. Factors such as droplet size influence droplet morphology and evaporation, thereby affecting ignition delay time [52]. Based on the experimental studies of Yuan L [53], it was found that when the spray hole diameter exceeds 0.75 mm, the possibility of forming a jet flow arises. To better simulate the actual leakage conditions of engine lubricating oil, needle diameters smaller than this were selected to ensure that the oil contacts the hot plate in droplet form. Under the same pressure, the larger the spray hole diameter, the larger the initial droplet diameter. The experimental results are shown in Fig 10.

As shown in Fig 10, the ignition delay time increases with the initial droplet diameter and is also influenced by the hot surface temperature. The experimental results align well with previous studies [54]. When the droplet diameters are 0.4 mm, 0.5 mm, 0.6 mm, and 0.7 mm, the ignition delay times range from 0.6–2.4 s, 1.8–3.4 s, 2.6–3.6 s, and 3.2–4.8 s, respectively. At a hot surface temperature of 370°C, the difference in ignition delay time becomes more pronounced with increasing droplet size. This is because, at relatively low surface temperatures, the heating phase dominates the evaporation process for smaller droplets. Larger droplets have longer lifetimes and thus longer ignition delay times [55]. Under the same temperature conditions, the ignition delay time of lubricating oil increases as the spray hole diameter becomes larger. This phenomenon can be attributed to the fundamental principle of hot surface ignition, where only a small portion of the combustible material undergoes chemical reactions when locally heated by the high-temperature ignition source, while the reaction rate in most of the material remains negligible. In the experiment, the injected droplets predominantly landed at the center of the hot plate. Due to the high viscosity and poor fluidity of engine lubricating oil, larger spray hole diameters produce larger droplets, which also result in increased spreading diameters. The deformation process of larger droplets is slower, leading to reduced heat transfer between the droplet and the plate. This reduction in heat transfer efficiency further decreases the droplet’s evaporation rate, thereby extending its lifetime and ultimately increasing the ignition delay time.Moreover, the larger droplet size alters the contact area and the dynamics of the thermal boundary layer, further reducing the droplet’s heat absorption efficiency. This indicates that, beyond geometric factors, the spray hole diameter significantly impacts ignition delay time by influencing droplet spreading behavior, heat transfer characteristics, and evaporation dynamics. Larger droplets require more time to reach equilibrium temperature and absorb the heat needed for evaporation, increasing the accumulation time required for the vapor concentration to reach ignition conditions [56]. Conversely, smaller droplets have a larger contact surface area relative to their volume when in contact with the hot surface, enhancing heat exchange and increasing the evaporation rate. This results in higher mixture concentrations and shorter ignition delay times (Table 6).

**Table 6:** Global average ignition delay time of oil droplets with diameter of 0.4-0.7 mm.

Oil volume/mm	0.4	0.5	0.6	0.7
Average ignition delay time/s	1.55	2.60	3.18	3.90

### Ignition probability and risk analysis of engine hot surface

**Fig 11:**
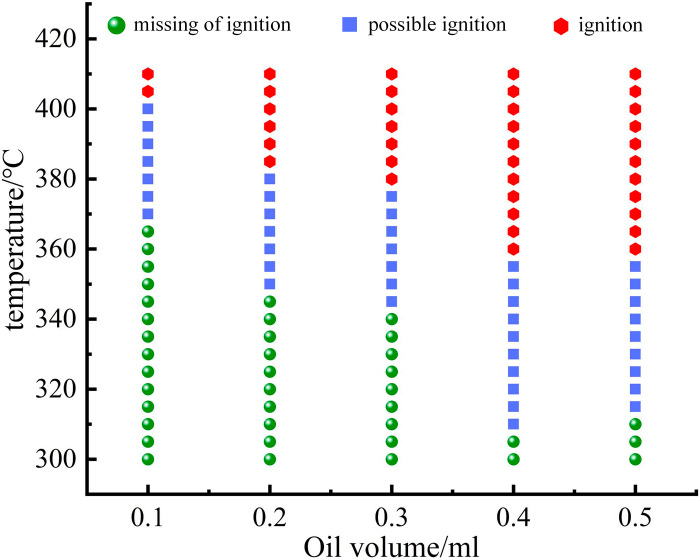
Relationship between Hot Surface Temperature and Ignition Event.

Hot surface ignition is defined as the appearance and propagation of a flame. In the initial test series for each sample, the temperature of the hot plate was increased in larger increments until hot surface ignition was observed, providing a rough estimate of the ignition temperature. At a determined hot surface temperature, three types of ignition events were recorded over 10 tests: no ignition (defined as non-ignition temperature), a mixture of ignition and non-ignition events (defined as possible ignition temperature), and ignition in all 10 tests (defined as percentage ignition temperature). Once the required number of ignition or non-ignition events was recorded at the initial surface temperature, the temperature was increased by approximately 5°C. At each subsequent surface temperature, the required ignition or non-ignition events were recorded to continue the testing sequence. When 10 consecutive ignition events were observed at a given surface temperature, the temperature was further increased by 5°C, and an additional set of 10 tests was conducted. If these resulted in another 10 consecutive ignition events, the higher temperature was recorded as the upper limit temperature for the test (ignition events were assigned a value of 1, while non-ignition events were assigned a value of 0). Additionally, the temperatures listed in this study represent the average of four measurements taken on the hot surface surrounding the droplet. After each droplet evaporated, regardless of whether ignition occurred, a blower was used to remove any residual gases around the hot surface. The stainless-steel plate was scrubbed with a steel wool pad and rinsed with ethanol to prevent residual substances from affecting subsequent experiments. The evolution of ignition probability for oil droplets ranging from 0.1 ml to 0.5 ml at different temperatures is shown in Fig 11.

**Fig 12:**
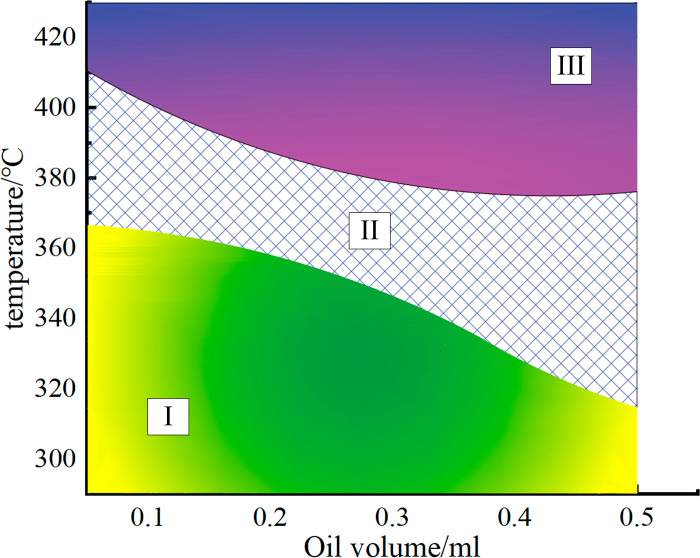
Risk Classification Diagram of Hot Surface.

Based on the experimental statistical results, data fitting was performed for the upper and lower boundaries of the temperature range for potential ignition areas, as shown in Fig 12. The fitted curve divides hot surface ignition events into three regions: I: Below the minimum ignition temperature curve. This area is a safe zone where no hot surface ignition events occur. II: Between the minimum ignition temperature curve and the 100% ignition temperature curve. This is the potential ignition zone, where fire hazards exist. The ignition probability increases with temperature, and the closer it is to the upper boundary, the higher the risk. III: Above the 100% ignition temperature curve. This area has the highest risk, and fire will inevitably occur once the conditions in this region are met.

A notable characteristic of the experimental data in this study is the overlap between ignition and non-ignition cases. Within this range, both hot surface ignition and non-ignition scenarios can occur, which warrants attention. The fitting equations for the upper and lower boundaries of the potential ignition zone have *R*^*2*^ values exceeding 95, indicating a good fit. As shown in Fig 12, with the increase in oil volume, the non-ignition range of the droplets gradually narrows, while the potential ignition range and inevitable ignition range expand. This indicates a certain correlation between oil volume and ignition risk: the larger the oil volume, the more the droplets tend to be “easier to ignite.” When the oil volume increases to 0.4 ml, this trend slows down. This phenomenon can be explained from two aspects: heat capacity and evaporation rate. First, there are differences in the heat capacity of oil droplets of different volumes. As the oil volume increases, the total heat capacity of the droplet significantly increases. This means that larger droplets can absorb more heat within the same heating duration. As a result, larger oil droplets can reach localized ignition conditions at lower temperatures, lowering the lower limit of the potential ignition temperature range (e.g., a 0.5 ml droplet may exhibit potential ignition at 315°C, whereas a 0.1 ml droplet requires 370°C). Second, there are significant differences in the evaporation rates of droplets with different volumes. Smaller droplets, due to their larger surface area-to-volume ratio, evaporate more quickly, making it difficult to accumulate sufficient combustible gases at lower temperatures. This means that inevitable ignition can only be achieved at higher temperatures, resulting in a narrower inevitable ignition range for smaller droplets. In contrast, larger droplets evaporate more slowly but can continuously release combustible gases at lower temperatures. As the temperature rises, the concentration of combustible gases rapidly accumulates to the level required for ignition, significantly reducing the lower limit of the potential ignition range and expanding the inevitable ignition range.

### Analysis of geometric characteristics of combustion flame of engine lubricating oil

**Fig 13:**
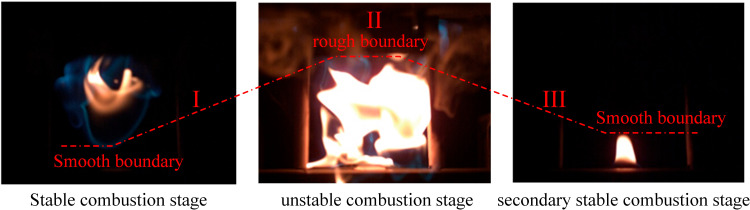
Three classic combustion stages of the flame.

From Fig 13, it can be seen that the combustion of the flame can be roughly divided into three stages: stable combustion phase (flame boundary is smooth), unstable combustion phase (flame boundary is rough), and secondary stable combustion phase (flame boundary returns to smooth). To quantify this pattern, high-speed cameras were used to capture the colored images of flames generated by droplets with volumes of 0.1–0.5 ml (fixed nozzle diameter of 0.4 mm) at 370°C, as well as droplets with a volume of 0.3 ml (fixed nozzle diameter of 0.4–0.7 mm). Using the SOBEL threshold segmentation algorithm and grayscale image binarization, the binary flame outer contour was obtained. Finally, the fractal dimension time-domain evolution characteristics of the flame from generation to extinction were determined using the box-counting method.

**Fig 14:**
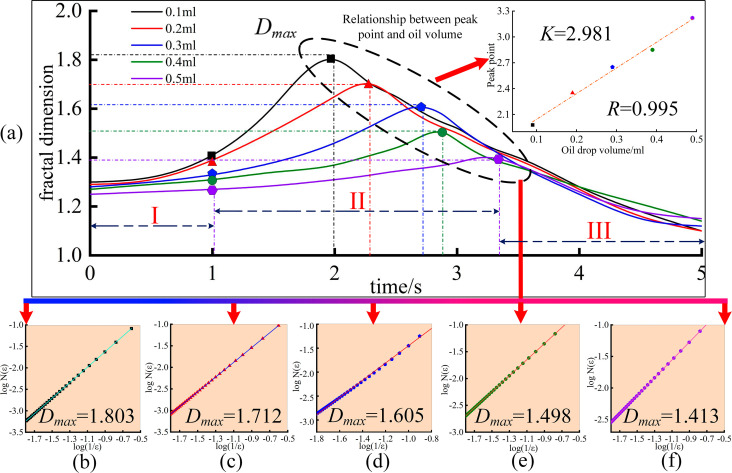
Evolution of flame fractal dimension of oil droplets with different volumes.

**Fig 15:**
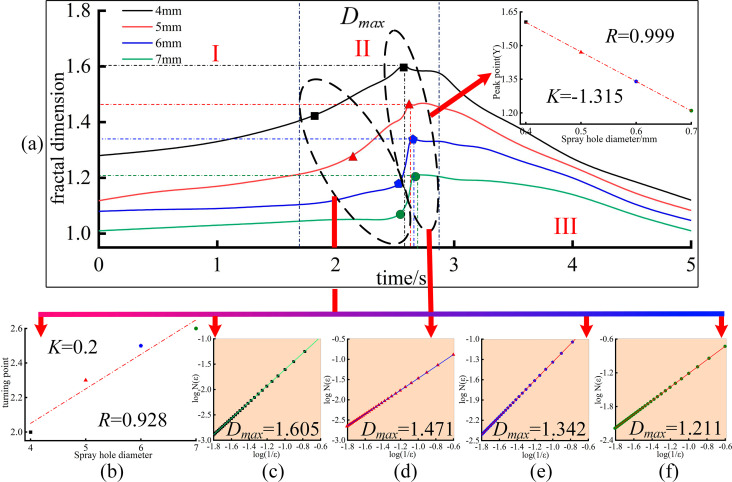
Fractal dimension evolution of oil droplet flame under different spray hole diameter.

Fig 14 shows the evolution of the fractal dimension within 5 seconds after the combustion of droplets with different volumes. Fig 15 shows the evolution of the fractal dimension within 5 seconds after the combustion of droplets with different nozzle diameters. A fractal dimension closer to 2 indicates that the flame’s edge is rougher and the geometry is more complex. Similarly, a fractal dimension closer to 1 indicates that the flame’s outer contour is smoother and the geometry is simpler.

As shown in Fig 14(b) to 14(c) and Fig 15(c) to 15(f), the grid number *N*(ε) required to cover the flame boundary exhibits a good linear relationship with *N*(1/ε) on the logarithmic scale, indicating that the flame from the lubricating oil combustion demonstrates clear fractal characteristics. This is similar to the study by Chatakonda [57], but with a key difference: the grid scale range in this study is limited to 1/2 (offering higher calculation precision), and the calculation range is broader, from 1/2 to 1/64, providing a higher level of generality. Analysis of Fig 14 (a) shows that within the first 1 second, the geometric shapes of flames generated by 0.1–0.5 ml of lubricating oil are relatively similar, and their fractal dimension curves do not exhibit significant divergence. After 1 second, the fractal dimension curves of flames for different droplet volumes show a marked upward trend, with the flame contours becoming increasingly complex as droplets transition to unstable combustion. The time to reach the fractal dimension peak for the droplet flames is linearly correlated with the droplet volume. For droplet volumes of 0.1, 0.2, 0.3, 0.4, and 0.5 ml, the combustion flame fractal dimension peaks occur at 1.98 s, 2.35 s, 2.65 s, 2.85 s, and 3.22 s, respectively. Fitting the relationship between time (*t*) and droplet volume (*V)* yields a linear correlation coefficient (*R* = 0.995, The *R*-value represents the Pearson correlation coefficient, which indicates the degree of linear correlation between two variables. The closer the absolute value of *R* is to 1, the stronger the linear correlation between the variables; the closer it is to 0, the weaker the correlation [58]) and (*P* = 0.001) (indicating a very low probability of this correlation occurring by chance for two unrelated variables). Therefore, the fractal dimension is significantly correlated with droplet volume. The fitted curve is shown as an inset in Fig 14 (a). Additionally, droplet volume (*V*) influences the fractal dimension peak (*D*_*max*_). For a 0.1 ml droplet, the flame fractal dimension peak is the highest (*D*_*max*_ = 1.803), with the slope of the fitted line in Fig 14 (b)). For a 0.5 ml droplet, the flame fractal dimension peak is the lowest (*D*_*max*_ = 1.413), with the slope of the fitted line in Fig 14 (f)). The same trend applies to Fig 14 (c), Fig 14 (d), and Fig 14 (e). Thus, droplet volume influences two critical parameters of the flame fractal dimension: the peak value and the time to reach the peak. During lubricating oil combustion, as droplet volume increases (0.1 to 0.5 ml), larger droplets exhibit greater thermal inertia, reducing internal temperature gradients and evaporation rates. This diminishes combustion irregularities, lowers the complexity of the flame interface, and decreases the fractal dimension peak. Simultaneously, larger droplets require more time to accumulate heat to reach the critical conditions for evaporation and combustion, resulting in a delayed occurrence of *D*_*max*_.

From Fig 15(a), it can be seen that the fractal dimension evolution of oil droplet flames under different spray hole diameters is generally similar to that of flames with varying droplet volumes: the fractal dimension curve grows slowly in the early stage (within 1 second), during which the flame burns relatively stable. The fractal dimension *D* remains below 1.4, and the flame’s outer contour is smooth and regular. From 1 to 2 seconds, the fractal dimension enters an unstable combustion transition period, rapidly increasing as the flame’s outer contour shifts from simple to complex. From 2 to 3 seconds is the peak fractal dimension period, during which all spray hole diameters reach the peak fractal dimension. After the peak, the fractal dimension *D* starts to decline, eventually approaching 1. This trend continues until the fifth second, when the flame disappears or its outer boundary can no longer be detected by the camera. Thus, combining the analysis of Fig 14 (a) and Fig 15 (a), it is evident that the fractal dimension evolution trend is overall consistent: a slow initial growth, followed by a rapid increase to the peak, and then a gradual decline toward 1. This corresponds to the combustion characteristics of lubricating oil: the flame burns steadily at the start, and when the droplet’s interior reaches the ignition point, the flame spreads rapidly, forming a complex boundary and reaching the most unstable combustion state. Finally, the residual small droplets inside the main droplet are ignited, but due to higher internal pressure, the flame’s expansion radius becomes limited and cannot influence the flame’s outer boundary. At this stage, the flame’s outer contour becomes increasingly stable, resulting in a gradual reduction in the geometric complexity of the flame’s outer boundary. This explains the absence of a secondary transition in the fractal dimension after the peak *D*_*max*_. However, closer observation reveals that the time at which the fractal dimension peak *D*_*max*_ occurs is not sensitive to spray hole diameter (for spray hole diameters of 0.4–0.7 mm, the times of *D*_*max*_ are 2.60 s, 2.62 s, 2.64 s, and 2.67 s, respectively). In contrast, the magnitude of the peak is more sensitive to spray hole diameter (as shown in the inset of Fig 15 (a), the fitted relationship between *D*_*max*_ and spray hole diameter *S* has a slope of *K*=−1.315). Additionally, spray hole diameter significantly affects the time at which the fractal dimension begins to increase rapidly. Droplets from a 0.4 mm spray hole transition first (at 2 seconds), while those from a 0.7 mm spray hole transition last (at 2.6 seconds). This linear relationship is shown in Fig 15 (b), with *R* = 0.928, confirming a significant correlation.

This study provides a crucial foundation for the practical application of fire prevention and control in vehicles. The temporal evolution analysis of flame fractal dimensions can be utilized for the early detection of fire hazards in automobiles, enabling rapid assessment of fire risk levels through real-time monitoring of flame geometric characteristics. Additionally, this method can be applied to optimize the design of automatic fire suppression systems in vehicles, allowing precise localization of fire sources and adjustment of the extinguishing agent’s spray range and intensity. Furthermore, the study of fractal dimensions can contribute to the thermal management design of high-temperature vehicle components, aiding in the development of more efficient cooling systems and protective materials, thereby significantly enhancing overall vehicle safety.

## Conclusions

This study utilized a self-developed automotive hot surface ignition oil simulation experimental platform to investigate the factors influencing the ignition delay time of engine lubricating oil, the ignition and risk characteristics of engine hot surfaces, and the time-domain evolution characteristics of the flame fractal dimension of engine lubricating oil. The main findings are as follows:

(1) As the hot surface temperature increases, the ignition delay time generally shows a decreasing trend, with 450°C being a critical turning point. Below 450°C, the ignition delay time decreases rapidly, and significant differences are observed between different oil volumes; above 450°C, the rate of decrease slows, and the ignition delay times for various oil volumes become more similar. Ignition delay time exhibits a clear positive correlation with single-injection oil volume, increasing with oil volume at 370°C. The ignition delay time also increases with the initial droplet diameter; at 370°C, for droplet diameters in the range of 0.4–0.7 mm, the ignition delay time ranges from 0.6 to 4.8 seconds.
(2) Ignition and non-ignition scenarios overlap within a specific range, forming a potential ignition zone. The fitted equations for its upper and lower boundaries have *R*^*2*^ values exceeding 95%, indicating excellent fitting performance. As the oil volume increases, the hot surface ignition temperature gradually decreases, but the rate of decrease significantly slows when the oil volume reaches 0.4 ml.
(3) The fractal dimension effectively quantifies the geometric complexity of the flame’s outer contour, thus characterizing the flame’s combustion stability. The evolution of the flame’s fractal dimension for lubricating oil droplets exhibits a trend of initial increase followed by gradual decrease (fluctuating in the range of 1–2). From 0 to 1 second is the stable combustion interval, during which the fractal dimension rises slowly. From 1 to 3 seconds is the unstable combustion interval, characterized by a sudden increase in the fractal dimension, reaching a peak *D*_*max*_, and then gradually declining. From 3 to 5 seconds is the secondary stable combustion interval, where the fractal dimension decreases slowly from the peak to near 1, and the flame’s outer contour transitions from complex to simple.
(4) The droplet volume *V* affects both the peak fractal dimension *D*_*max*_ of the flame and the time *t*_*max*_ at which it occurs. Larger droplet volumes result in the fractal dimension peak occurring later. For a 0.1 ml droplet, *D*_*max*_ appears the earliest (*t*_*max*_ = 1.98 s), while for a 0.5 ml droplet, *D*_*max*_ appears the latest (*t*_*max*_ = 3.22 s). There is a significant correlation between *t*_*max*_ and *V* (*R* = 0.995, *P* = 0.001). Spray hole diameter has a greater impact on the magnitude of *D*_*max*_ than *t*_*max*_. For spray hole diameters ranging from 0.4 mm to 0.7 mm, the fractal dimension peaks for all droplets appear around 2.6 seconds, but the magnitude of *D*_*max*_ varies significantly. Smaller spray hole diameters result in larger *D*_*max*_. At a spray hole diameter of 0.4 mm, *D*_*max*_ is the largest, and the geometric changes in the flame’s outer contour are the most dramatic, indicating the most unstable combustion process overall.

## Supporting information

S1 Data Data disclosure (Bai - manuscript). (RAR)

## References

[1] Nomikos P, Rahmani R, Morris N, Rahnejat H. An investigation of oil leakage from automotive driveshaft radial lip seals. Proceedings of the Institution of Mechanical Engineers, Part D: Journal of Automobile Engineering. 2022;237(13):3108–24. doi: 10.1177/09544070221127105

[2] Murthy AA, Norris S, Subiantoro A. Experimental investigation of internal leakages and effects of lubricating oil on the performance of a four-intersecting-vane rotary expander. Energy. 2022;238:121689. doi: 10.1016/j.energy.2021.121689

[3] Deng J, Yang W, Zhang YN, Chen J, Li Y, Ji X, et al. Experimental study on hot surface ignition and flame characteristic parameters of lubricating oil. Journal of Thermal Analysis and Calorimetry. 2024;116:1–13.

[4] Ma Q, Zhang C, Ren H. Application of bayesian network in the investigation of vehicle spontaneous combustion accident. Advances in Transportation Studies. 2014.

[5] Egorov RI, Antonov DV, Valiullin TR, Strizhak PA. The ignition dynamics of the water-filled fuel compositions. Fuel Processing Technology. 2018;174:26–32. doi: 10.1016/j.fuproc.2018.02.003

[6] Vershinina KYU, Dorokhov VV, Romanov DS, Strizhak PA. Comparing the ignition parameters of promising coal fuels. Process Safety and Environmental Protection. 2020;139:273–82. doi: 10.1016/j.psep.2020.04.027

[7] Jiang Y-H, Li G-X, Li H-M, Li L, Zhang G-P. Effect of flame inherent instabilities on the flame geometric structure characteristics based on wavelet transform. International Journal of Hydrogen Energy. 2018;43(18):9022–35. doi: 10.1016/j.ijhydene.2018.03.141

[8] Liu J, Zhang X, Xie Q. Flame geometrical characteristics of downward sloping buoyant turbulent jet fires. Fuel. 2019;257:116112. doi: 10.1016/j.fuel.2019.116112

[9] Roy A, Sujith RI. Fractal dimension of premixed flames in intermittent turbulence. Combustion and Flame. 2021;226:412–8. doi: 10.1016/j.combustflame.2020.12.032

[10] El-Nabulsi RA, Anukool W. Modeling thermal diffusion flames with fractal dimensions. Thermal Science and Engineering Progress. 2023;45:102145. doi: 10.1016/j.tsep.2023.102145

[11] Glushkov DO, Legros J-C, Strizhak PA, Volkov RS. Heat and mass transfer at the ignition of vapors of volatile liquid fuels by hot metal core: Experimental study and modelling. International Journal of Heat and Mass Transfer. 2016;92:1182–90. doi: 10.1016/j.ijheatmasstransfer.2015.09.087

[12] Stouffer SD. Fuel effects on altitude relight performance of a swirl cup combustor. AIAA Scitech 2020 Forum. 2020;2020:1822.

[13] Mével R, Melguizo-Gavilanes J, Boeck LR, Shepherd JE. Experimental and numerical study of the ignition of hydrogen-air mixtures by a localized stationary hot surface. International Journal of Heat and Fluid Flow. 2019;76:154–69. doi: 10.1016/j.ijheatfluidflow.2019.02.005

[14] Boeck LR, Melguizo-Gavilanes J, Shepherd JE. Hot surface ignition dynamics in premixed hydrogen–air near the lean flammability limit. Combustion and Flame. 2019;210:467–78. doi: 10.1016/j.combustflame.2019.09.002

[15] Zhang S, Gogos G. Film evaporation of a spherical droplet over a hot surface: fluid mechanics and heat/mass transfer analysis. J Fluid Mech. 1991;222(1):543. doi: 10.1017/s0022112091001210

[16] Michael Bennett J. Ignition of combustible fluids by heated surfaces. Process Safety Progress. 2001;20(1):29–36. doi: 10.1002/prs.680200107

[17] Gulder O. Spheroidal evaporation and ignition of fuel droplets on a hot surface. Symposium (International) on Combustion. 1985;20(1):1751–60.

[18] Colwell JD, Reza A. Hot surface ignition of automotive and aviation fluids. Fire Technology. 2005;41(2):105–23.

[19] Kuchta JM, Bartkowiak A, Zabetakis MG. Hot Surface Ignition Temperatures of Hydrocarbon Fuel Vapor-Air Mixtures. J Chem Eng Data. 1965;10(3):282–8. doi: 10.1021/je60026a023

[20] Davis S, Chavez D, Kytomaa H. Hot surface ignition of flammable and combustible liquids. SAE Technical Paper. 2006;2006.

[21] Davis S, Kelly S, Somandepalli V. Hot Surface Ignition of Performance Fuels. Fire Technol. 2009;46(2):363–74. doi: 10.1007/s10694-009-0082-z

[22] Goyal V, Benhidjeb Carayon A, Simmons R, Meyer S, Gore JP. (2017). Hot surface ignition temperatures of hydrocarbon fuels[C]. In 55th AIAA Aerospace Sciences Meeting. 2017;2017:0826.

[23] Lapointe NR, Adams CT, Washington J. Hot surface ignition of gasoline on engine materials. World Congress & Exhibition. 2006.

[24] Byers K, Epling W, Cheuk FK. Evaluation of automobile fluid ignition on hot surfaces. SAE Transactions. 2007;131:1312–7.

[25] Mitu M, Brandes E, Hirsch W. Ignition temperatures of combustible liquids with increased oxygen content in the (O2 + N2) mixture. Journal of Loss Prevention in the Process Industries. 2019;62:103971. doi: 10.1016/j.jlp.2019.103971

[26] Shaw A, Epling W, McKenna C, Weckman B. Evaluation of the Ignition of Diesel Fuels on Hot Surfaces. Fire Technol. 2009;46(2):407–23. doi: 10.1007/s10694-009-0098-4

[27] Huang Z, Kan W, Lu Y, Cheng T, Yu L, Hu X. Effect of Nanoparticle Suspensions on Liquid Fuel Hot-Plate Ignition. Journal of Nanotechnology in Engineering and Medicine. 2014;5(3):. doi: 10.1115/1.4029029

[28] Kumar N, Tomar M. Influence of nanoadditives on ignition characteristics of Kusum ( Schleichera oleosa ) biodiesel. Int J Energy Res. 2019;43(8):3223–36. doi: 10.1002/er.4446

[29] Chybowski L. Study of the Relationship between the Level of Lubricating Oil Contamination with Distillation Fuel and the Risk of Explosion in the Crankcase of a Marine Trunk Type Engine. Energies. 2023;16(2):683. doi: 10.3390/en16020683

[30] Law CK, Sung CJ. Structure, aerodynamics, and geometry of premixed flamelets. Progress in Energy and Combustion Science. 2000;26(4–6):459–505. doi: 10.1016/s0360-1285(00)00018-6

[31] Gollner MJ, Miller CH, Tang W, Singh AV. The effect of flow and geometry on concurrent flame spread. Fire Safety Journal. 2017;91:68–78. doi: 10.1016/j.firesaf.2017.05.007

[32] Yokev N, Greenberg JB. Linear stability analysis of laminar premixed water-in-fuel emulsion spray flames. Fuel. 2018;222:733–42. doi: 10.1016/j.fuel.2018.02.113

[33] Wang C, Zhang X, Huang Y, Xie J, Xu M, Zhang J. Flame expansion behavior induced by a water droplet impacting on burning and stratified oil-water pool. International Journal of Thermal Sciences. 2023;191:108342. doi: 10.1016/j.ijthermalsci.2023.108342

[34] Wardana ING. Combustion characteristics of jatropha oil droplet at various oil temperatures. Fuel. 2010;89(3):659–64. doi: 10.1016/j.fuel.2009.07.002

[35] Klein M, Herbert A, Kosaka H, Böhm B, Dreizler A, Chakraborty N, et al. Evaluation of Flame Area Based on Detailed Chemistry DNS of Premixed Turbulent Hydrogen-Air Flames in Different Regimes of Combustion. Flow Turbulence Combust. 2019;104(2–3):403–19. doi: 10.1007/s10494-019-00068-2

[36] Zhuang H, Hung D, Xu M, Chen H, Li T, Zhang Y. Flame area correlations with heat release at early flame development of combustion process in a spark-ignition direct-injection engine using gasoline, ethanol and butanol. 2013.

[37] Luo S, Xu J, Wang C, Ji J. Experimental study on the dynamic evolution behavior and heat transfer of flame spread over continuously flowing diesel fuel. International Communications in Heat and Mass Transfer. 2024;159:108287. doi: 10.1016/j.icheatmasstransfer.2024.108287

[38] Hagerhall CM, Purcell T, Taylor R. Fractal dimension of landscape silhouette outlines as a predictor of landscape preference. Journal of Environmental Psychology. 2004;24(2):247–55. doi: 10.1016/j.jenvp.2003.12.004

[39] Rezaie A, Mauron AJP, Beyer K. Sensitivity analysis of fractal dimensions of crack maps on concrete and masonry walls. Automation in Construction. 2020;117:103258. doi: 10.1016/j.autcon.2020.103258

[40] Cao T, Wang W, Tighe S, Wang S. Crack image detection based on fractional differential and fractal dimension. IET Computer Vision. 2019;13(1):79–85. doi: 10.1049/iet-cvi.2018.5337

[41] Torabian M, Pourghassem H, Mahdavi-Nasab H. Fire Detection Based on Fractal Analysis and Spatio-Temporal Features. Fire Technol. 2021;57(5):2583–614. doi: 10.1007/s10694-021-01129-7

[42] Ai T, Zhang R, Zhou HW, Pei JL. Box-counting methods to directly estimate the fractal dimension of a rock surface. Applied Surface Science. 2014;314:610–21. doi: 10.1016/j.apsusc.2014.06.152

[43] Distaso E, Amirante R, Calò G, De Palma P, Tamburrano P, Reitz RD. Predicting lubricant oil induced pre-ignition phenomena in modern gasoline engines: The reduced GasLube reaction mechanism. Fuel. 2020;281:118709. doi: 10.1016/j.fuel.2020.118709

[44] El-Sharkawy A, Sami A, Hekal AE-R, Arora D, Khandaker M. Transient Modelling of Vehicle Exhaust Surface Temperature. SAE Int J Mater Manf. 2016;9(2):321–9. doi: 10.4271/2016-01-0280

[45] Boyarshinov MG, Kuznetsov NI. Thermal regime of automobile exhaust system at low temperature. World of Transport. 2019;17(4):83.

[46] Wang Z, Chen J, Yu Y, Kong D. Experimental study on the ignition and burning characteristics of liquid fuels on hot surfaces. Process Safety and Environmental Protection. 2023;176:725–33. doi: 10.1016/j.psep.2023.06.050

[47] Yu S, Yin B, Deng W, Jia H, Ye Z, Xu B, et al. Internal flow and spray characteristics for elliptical orifice with large aspect ratio under typical diesel engine operation conditions. Fuel. 2018;228:62–73. doi: 10.1016/j.fuel.2018.04.156

[48] Bohra LK, Mincks LM, Garimella S. Experimental investigation of pressure drop characteristics of viscous fluid flow through small diameter orifices. Journal of Fluids Engineering. 2021;143(2):021306.

[49] Zhang W, Yu T, Fan J, Sun W, Cao Z. Droplet impact behavior on heated micro-patterned surfaces. Journal of Applied Physics. 2016;119(11):. doi: 10.1063/1.4943938

[50] Bennett JM, Ballal D. Ignition of combustible fluids by heated surfaces. 41st Aerospace Sciences Meeting and Exhibit. n.d.

[51] Xiong TY, Yuen MC. Evaporation of a liquid droplet on a hot plate. International Journal of Heat and Mass Transfer. 1991;34(7):1881–94. doi: 10.1016/0017-9310(91)90162-8

[52] Labeish VG, Pimenov AG. Experimental study of heat transfer between a hot wall and impinging drops. Journal of Engineering Physics. 1984;47(6):1400–6. doi: 10.1007/bf00870055

[53] Yuan L. Ignition of hydraulic fluid sprays by open flames and hot surfaces. Journal of Loss Prevention in the Process Industries. 2006;19(4):353–61. doi: 10.1016/j.jlp.2005.09.001

[54] Long W, Yi P, Jia M, Feng L, Cui J. An enhanced multi-component vaporization model for high temperature and pressure conditions. International Journal of Heat and Mass Transfer. 2015;90:857–71. doi: 10.1016/j.ijheatmasstransfer.2015.07.038

[55] Yi P, Long W, Feng L, Wang W, Liu C. An experimental and numerical study of the evaporation and pyrolysis characteristics of lubricating oil droplets in the natural gas engine conditions. International Journal of Heat and Mass Transfer. 2016;103:646–60. doi: 10.1016/j.ijheatmasstransfer.2016.07.084

[56] Ma L, Qiu X, Zheng Z, Cui Y, Duan X. Numerical research on influencing factors of droplet evaporation in high temperature airflow. Petrochemical Technology. 2013;42(08):886–90.

[57] Chatakonda O, Hawkes ER, Aspden AJ, Kerstein AR, Kolla H, Chen JH. On the fractal characteristics of low Damköhler number flames. Combustion and Flame. 2013;160(11):2422–33. doi: 10.1016/j.combustflame.2013.05.007

[58] Zhang T, Li J, Ji X, Ji B, Feng G, Pan H, et al. Coherence analysis of the crack strain field in coal rock with borehole-crack composite defects. Theoretical and Applied Fracture Mechanics. 2024;133:104497. doi: 10.1016/j.tafmec.2024.104497

